# Non-destructive estimation of SPAD and biomass in *Lamiophlomis rotata* using hyperspectral imaging and deep learning with DRSA-CARS feature selection

**DOI:** 10.3389/fpls.2025.1640779

**Published:** 2025-09-18

**Authors:** Xuemei Wu, Liwen Zhong, Rong Ding, Chenghui Wang, Hongchuan Chen, Shihong Zhong, Rui Gu

**Affiliations:** ^1^ School of Ethnic Medicine, Chengdu University of Traditional Chinese Medicine, Chengdu, China; ^2^ State Key Laboratory of Southwestern Chinese Medicine Resources, School of Pharmacy, Chengdu University of Traditional Chinese Medicine, Chengdu, China; ^3^ School of Pharmacy, Southwest Minzu University, Chengdu, China

**Keywords:** hyperspectral data, time-series phenotypes, vegetation index, textural features, deep learning

## Abstract

**Introduction:**

Monitoring the growth status and aboveground biomass of wild and cultivated medicinal herbs remains a persistent challenge in precision agriculture.

**Methods:**

In this study, we developed machine learning and deep learning models to estimate SPAD values and biomass of *Lamiophlomis rotata* (Benth.). The models used hyperspectral data and time-series phenotypic traits from 508 samples collected across different altitudes. Regions of interest (ROIs) were manually defined from plant contours. The corresponding mean spectral profiles were then preprocessed. To improve feature selection, we proposed a Dynamic Reptile Search Algorithm-enhanced CARS (DRSA-CARS) method. This method integrates a dynamic behavioral strategy into the CARS framework to identify informative spectral bands. Vegetation indices (VIs) and gray-level co-occurrence matrix (GLCM)-based texture parameters were extracted and combined with spectral features to construct the PLSR, SVR, FNN, and CNN models.

**Results:**

Compared to CARS, the DRSA-CARS method reduced feature dimensionality by up to 75.7% for SPAD and 29.2% for biomass, while improving prediction accuracy (*R*²) by 24.4% and 34.7%, respectively. Among all models, the FNN achieved the highest performance, with *R*² values of 0.7732 (training) and 0.7502 (testing) for SPAD and 0.8260 and 0.7933 for biomass. Feature fusion further improved predictive accuracy by 11% for SPAD and 30% for biomass compared to models based on individual feature types.

**Discussion:**

These results demonstrate that coupling DRSA-CARS-based feature selection with deep learning provides a robust, non-destructive approach for evaluating plant growth status. This framework highlights the potential of hyperspectral imaging as a rapid, reliable, non-invasive tool for precision cultivation of medicinal herbs.

## Graphical Abstract

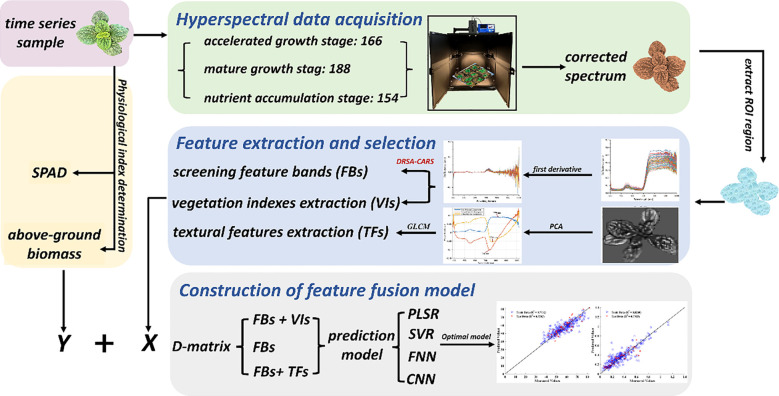

## 1 Introduction

The Nobel Prize awarded to Youyou Tu for the discovery of artemisinin from the medicinal plant *Artemisia annua* L. has reaffirmed the pivotal role of herbal medicine in alleviating human suffering and driving new drug discovery. In recent years, the global demand for herbal medicine has increased significantly, with annual consumption exceeding four million tons (Gao et al., 2020). To address the shortage of wild resources and the labor-intensive nature of harvesting, more than 200 species of herbal plants have been successfully cultivated (Zhang, 2019). Compared with other botanicals, alpine medicinal plants inhabit high-altitude environments, leading to harvesting challenges and limited resource availability (Nagy and G., 2009). Therefore, there is an urgent need to conduct cultivation trials, particularly for medicinal plants that grow at high altitudes and are widely used in clinical practice. However, numerous challenges remain in cultivation, with the quality of medicinal materials, such as phenotypic traits, chemical composition, and clinical efficacy, being a primary concern (Canter et al., 2005; Hao and Xiao, 2018).

Phenotype is a primary and intuitive indicator for evaluating the structural and functional characteristics of plants, and it can be used to identify varieties, assess quality, and monitor growth stages, among other applications (Feng et al., 2021). Dong et al. (2024) investigated three varieties of *Corydalis yanhusuo* collected from Zhejiang Province to screen for superior provenances based on phenotypic analyses of flower morphology and rhizome yield. The formation of drumstick-shaped roots, a distinctive phenotypic trait of *Panax notoginseng*, is commonly attributed to saponin accumulation and has been shown to be closely associated with carbohydrate metabolism in the plant’s vegetative organs (Zhang et al., 2022a). Another typical example of phenotypic variation with distinct functional implications is the comparison between Ziqin and Kuqin. Ziqin is characterized by a compact root interior, whereas Kuqin exhibits a hollow root structure. Both types are derived from *Scutellaria baicalensis* (Sun et al., 2023). Hence, the phenotype is considered a three-dimensional expression of space, time, and the interaction between genes and the environment. Importantly, the phenotype is tightly related to biomass, particularly in medicinal plants where the aboveground parts are utilized, an aspect of major concern for cultivators. Therefore, establishing a fast and real-time method for phenotypic monitoring is of great significance for quality evaluation and cultivation of medicinal plants.

Compared with traditional destructive analytical methods, spectral technology mitigates limitations such as labor intensity and chemical reagent consumption and has been widely applied to distinguish origins, processing methods, and adulteration, among others (Liu et al., 2023; Wu et al., 2018a, b). Hyperspectral imaging (HSI), in particular, is a non-destructive sensing technology that has gained popularity for real-time vegetation monitoring (Shakoor et al., 2017). Specifically, HSI can simultaneously capture hundreds of images across different wavelengths, providing detailed spectral signatures (Wang et al., 2021). Variations in leaf characteristics caused by environmental factors or growth stages can be captured in a timely manner by HSI. Consequently, HSI in combination with computational modeling has been widely used in agriculture to detect both external and internal quality attributes (Khan et al., 2022). For example, the internal quality of Red Globe grapes has been successfully estimated based on HSI spectral information and images collected at various growth stages. The correlation coefficients of the partial least squares regression (PLSR) model for the calibration and prediction sets were 0.9775 and 0.9762, respectively (S. Gao and Xu, 2022a). Additionally, vegetation indices (VIs) derived from maize at different growth stages using unmanned aerial vehicles (UAVs) have been used to explore chlorophyll content variation. The partial least squares (PLS) model for chlorophyll content prediction, constructed using optimal spectral variables and VIs, achieved *R*² values of 0.7530 and 0.682 for the training and validation sets, respectively (Qiao et al., 2022). The aboveground biomass of spinach crops grown under two different conditions was predicted using satellite imagery (Marcone et al., 2024). Despite these advances, HSI data are often high-dimensional and contain redundant spectral bands, which can hinder efficient modeling and reduce prediction robustness. This makes feature selection essential for extracting the most informative spectral variables for accurate and stable analysis.

Feature selection (FS) is a critical step in HSI to address high dimensionality, band collinearity, and noise, and retain discriminative information. FS methods are generally classified into filter, wrapper, and embedded approaches (Hennessy et al., 2020). Recent HSI studies have explored metaheuristic band selection schemes, including wild-horse optimizer variants and multimodal evolutionary strategies for unsupervised selection (Chen et al., 2024; Li et al., 2011). These approaches illustrate the rapid development of FS research. The Reptile Search Algorithm (RSA) is a swarm-intelligence optimizer inspired by crocodile behavior (Abualigah et al., 2022). It alternates between encircling (exploration) and hunting (exploitation) to balance global and local search and has demonstrated strong performance on high-dimensional optimization tasks. Classical RSA can prematurely converge and lose population diversity when solving complex, multimodal problems (Wu et al., 2023a). These issues become more severe in HSI feature selection, where bands are highly correlated and noisy. Recent studies have introduced improved RSA variants that add adaptive parameters, hybrid moves, or mutation to strengthen early exploration, enhance late-stage exploitation, and maintain diversity, thereby achieving more stable convergence (Khan et al., 2023). For wavelength selection, Competitive Adaptive Reweighted Sampling (CARS) combines Monte Carlo sampling with PLS coefficients to iteratively eliminate uninformative variables. This process produces parsimonious models, but its reliance on stochastic resampling and regression-coefficient paths can make CARS unstable across runs, sensitive to noise and sample size, and prone to discarding correlated but complementary bands (Li et al., 2009; Park et al., 2025). These limitations have led to stability-oriented extensions. To address these issues, we embed a dynamic RSA (DRSA) into the CARS framework to stabilize and optimize variable elimination in noisy, high-dimensional HSI. This approach produces robust and compact band subsets for real-time phenotyping and biomass estimation. Collectively, DRSA-CARS combines CARS’s parsimony with dynamic global search to improve stability against run-to-run variability and noise, broadening its applicability to medicinal plant HSI analysis. With these informative bands identified, integrating HSI with machine learning (ML) offers powerful tools for modeling target variables and enhancing prediction performance.

ML, one of the fastest-growing technical fields, currently forms the core of artificial intelligence and data science (Jordan and Mitchell, 2015). ML primarily includes unsupervised learning, supervised learning, and neural networks (Mahesh, 2020). The integration of HSI and ML has proven effective in predicting target variables. However, the suitability of each method depends on the specific algorithm and the nature of the data problem. Numerous studies have combined HSI with ML methods to effectively assess the quality and yield of medicinal plants. HSI data processed with multiplicative scatter correction and modeled with Bayesian ridge regression have been used to evaluate flavonoid content in *Ginkgo biloba* leaves, achieving an *R*² value of 0.8700 for the test set (Lu et al., 2024). This study demonstrates a rapid and accurate approach for assessing the quality of *G. biloba* leaves. Additionally, biomass of *Mentha* crops was estimated using spectral data and a multilayer perceptron artificial neural network (ANN) model, yielding an *R*² value of 0.7620 (Khan et al., 2020). Therefore, the integration of non-destructive, environmentally friendly, and rapid spectroscopic techniques with ML provides a promising approach for evaluating the quality and biomass of cultivated medicinal plants.

*Lamiophlomis rotata* (Benth.) Kudo is a perennial medicinal plant widely distributed across meadows, grasslands, and gravelly habitats at altitudes ranging from 2,700 to 4,500 m (Ding et al., 2023). *Lamiophlomis rotata* has been traditionally used for its medicinal properties, including the treatment of injuries, relief of muscle and bone pain, reduction of joint swelling, and management of conditions such as dysmenorrhea and metrorrhagia (Cui et al., 2020). In the herbal medicine market, most *L. rotata* is sourced from wild populations. Due to increasing demand and overharvesting, *L. rotata* has become endangered and is now classified as a first-class endangered Tibetan medicinal plant (Li et al., 2021). Therefore, artificial cultivation is necessary to promote the survival and sustainable utilization of *L. rotata*. However, various environmental uncertainties, such as changes in altitude, climate variability, and insect infestations, can affect the growth and development of *L. rotata* during artificial cultivation. Liu et al. (2020) found that leaf phenotypes, including length, width, and thickness, exhibited plasticity along an altitudinal gradient ranging from 3,000 to 4,600 m. Changes in leaf shape and thickness may result in altered biomass allocation patterns and metabolic pathways. In previous studies, destructive analytical methods have been used to investigate the metabolic responses of wild *L. rotata* collected from different altitudes (Ma et al., 2015). However, such invasive methods are not suitable for real-time evaluation of the growth status of cultivated *L. rotata*.

In view of this, the objectives of this study are as follows: 1) to investigate the temporal phenotypic variations of *L. rotata* grown at three different altitudes; 2) to evaluate the potential of an HSI system to monitor the vegetative growth of cultivated *L. rotata* with Soil Plant Analysis Development (SPAD) values and aboveground biomass as references; 3) to compare the effectiveness of wavelength selection between the proposed DRSA-CARS method and the classical CARS on HSI data; and 4) to compare the predictive performance of regression models based on individual spectral features and on fused features, including VIs and textural features (TFs). The ultimate goal is to develop a rapid, non-invasive, and efficient HIS-based method integrated with deep learning models to predict vegetation dynamics and aboveground biomass of *L. rotata*.

## 2 Materials and methods

### 2.1 Experimental materials

In this study, *L. rotata* materials were collected from Sichuan Province with three altitude districts, and the detailed information is summarized in **Table 1**. The average temperature and humidity were supervised in real time by the Jingxun yun platform (Weihai Jingxun Unimpeded Electronic Technology Co., Ltd., Jinan Branch). The sowing and transplanting experiments of *L. rotata* were conducted at the Sichuan Hongyuan Endangered Alpine Medicinal Plants Breeding Technology Center. To exclude the influence of soil factors and the interaction among *L. rotata* plants, one plant was transplanted into a flowerpot measuring 11cm in height and 9cm in width on 21 March 2024, and gap filling was conducted on 5 May 2024. Throughout the experiment, the irrigation procedure, fertilizer schedule, and pest and disease management procedures were consistently maintained. A total of 720 (240 × 3) *L. rotata* plants were randomly selected based on the approximate uniform sizes and evenly allocated to three altitude districts (Hongyuan County, Qiongxi Town; Maoxian County, Shaba Town; and Chongzhou City, Jiguanshan Town) on 26 and 27 May 2024. Qiongxi (QX) and Shaba (SB) are located in the Aba Tibetan and Qiang Autonomous Prefecture of Sichuan Province, while Jiguanshan (JGS) is located in Chongzhou City, Sichuan Province. To consider the potential variability of feature importance across growth stages, the samples were collected at three representative time points, corresponding to early growth, rapid growth, and maturity stages of *L. rotata* at each altitude. These time points allow us to investigate whether the predictive contribution of spectral, textural, and vegetation index features remains consistent or varies over time. After 1 month of acclimation to growth conditions, some of the *L. rotata* samples died and then the cultivated *L. rotata* plants were collected from three separate altitudes, and the detailed sample information is shown in **Table 1**. In total, 508 samples were collected for this study.

**Table 1:** The cultivation and sample information of *Lamiophlomis rotata*.

District	Altitude	Longitude	Latitude	Sampling time	Sample amount	Monthly mean temperature	Monthly mean humidity
Qiongxi (QX)	3,506	102°34′09.1450″E	32°49′11.8074″N	June 25th	58	13.01	71.60
				July 15th	52	15.54	84.30
				August 6th	20	20.86	84.16
Shaba (SB)	2,560	103°31′14.7936″E	31°46′58.1124″N	June 24th	72	16.35	77.35
				July 14th	72	18.35	87.69
				August 5th	72	22.46	89.14
Jiguanshan (JGS)	1,293	103°20′56.1076″E	30°45′38.0419″N	June 26th	36	19.91	69.55
				July 16th	64	20.71	78.64
				August 7th	62	23.54	87.38

### 2.2 Data collection of hyperspectral images

In this program, a HY-6010-S NIR-HSI portable push broom hyperspectral system (Hangzhou Hyperspectral Imaging Technology Co., Ltd., China) equipped with an imaging spectrograph was used to collect hyperspectral images of *L. rotata* across three periods. This system consists of two 450-W halogen light sources, and the two lamps were set with the incident angle of the light source of 45° to provide uniform lighting in the field of view. The HSI image acquired by this system consists of 300 spectral bands (bit depth of 12) between 380 and 1,022 nm with a spectral resolution of 2.8 nm. The distance from the sensor to the samples was approximately 46cm, resulting in a spatial resolution of 25 µm per pixel, and the optimized frames per second (fps) was set to 5. After image acquisition, the raw spectra were radiometrically calibrated to relative reflectance using the HHIT software (version 1.9.1; Hangzhou Hyperspectral Imaging Technology Co., Ltd., China). Calibration was performed by subtracting dark-current noise and normalizing each spectrum to a 20% diffuse reflectance standard panel (nominal accuracy ± 2%), thereby reducing background noise and compensating for instrument response. Then, the radiation correction hyperspectral data were imported into ENVI 5.3 software (Research Systems Inc., Boulder, CO, USA) for further analysis.

### 2.3 ROI selection from hyperspectral images

With the advancement of image processing, the concept of region of interest (ROI) gradually emerged in fields such as computer vision and signal processing to facilitate more efficient and targeted data analysis. After radiometric calibration, the hyperspectral data were saved in SPE file format and imported into ENVI 5.6 software to extract the ROI of the *L. rotata* manually. In this study, the ROI was defined by drawing shapes based on the outline of the plant image. After the extraction of ROI, the mean spectra and spectral image were extracted, with the former saved as a data matrix of 300 wavelength bands and the latter saved as a metadata file HDR for subsequent analysis.

### 2.4 Measurement of phenotypic data

#### 2.4.1 Measurement of SPAD

The SPAD value is commonly used to estimate the chlorophyll content in plant leaves (Jiang et al., 2017). The relative index was used to assess the plant growth status and nitrogen levels. In this study, a non-destructive handheld chlorophyll meter (Konica Minolta SPAD-502Plus, Japan) was used to assess the chlorophyll content of *L. rotata.* To ensure data reliability, two opposite leaves were selected to obtain the SPAD value for each sample, and this data acquisition procedure was carried out at the cultivation site between 10a.m. and 12 p.m. on the sampling day. Finally, the average value calculated from both leaves of each *L. rotata* plant was considered the final valid data.

#### 2.4.2 Measurement of biomass

After HSI analysis, all *L. rotata* plants were removed from the flowerpots, and the surface soil both in the leaves and roots was washed off using running water. Subsequently, the *L. rotata* plants were separated into above- and belowground tissues, which were then dried at 60°C in a constant temperature oven (Experimental Instrument Factory, Shanghai, China) until constant weight was achieved. Afterward, the biomass of the aboveground tissue was accurately weighed using an electronic analytical balance (G&G JJ223BC, America).

### 2.5 Data processing and analysis

#### 2.5.1 Preprocessing of spectra

To eliminate interference from the environment and equipment, pretreatment methods such as standard normal variate (SNV), multiplicative scatter correction (MSC), Savitzky–Golay smoothing (SG), and first-order derivative (FD) were applied to process the raw spectra. SNV and MSC are commonly used to correct spectral errors caused by scattering among samples (Wu et al., 2018a). SG can preserve the details of the spectral signal by fitting it with a polynomial (Wu et al., 2018b). A derivative is usually employed to enhance the resolution and amplify the difference in the spectral signal, which has been mainly used for extracting fine structure features of HSI (Liu and Han, 2017). In this part, the best spectra preprocessing methods were evaluated by a PLSR model with a fixed parameter of components of 5 and a ratio of 7:2:1 of the training, validation, and test sets.

#### 2.5.2 Selection of feature wavelengths

The preprocessed spectral data matrix contains comprehensive information derived from HSI. Therefore, it is essential to apply effective feature selection techniques to reduce data redundancy and computational complexity. In particular, identifying the most informative wavelengths facilitates a clearer understanding of their contributions to model performance and enhances the explainability of spectral-based predictions. In this study, a widely applied technique in the field of feature band selection of the CARS algorithm was adopted as the primary method for selecting optimal feature bands associated with SPAD and aboveground biomass (Li et al., 2014; Su et al., 2021). Specifically, CARS selects features based on the absolute values of the regression coefficients in a PLS model and iteratively eliminates variables with lower contributions. The subset yielding the minimum root mean square error of cross-validation (RMSECV) is retained as the optimal band combination (Su et al., 2021; Yao et al., 2021; Zhang et al., 2021). However, despite its effectiveness in reducing data dimensionality, the classical CARS algorithm exhibits limitations in selecting the proper number of selected wavelengths. It focuses on maximizing prediction performance, but often overlooks the redundancy or multicollinearity among selected wavelengths.

To overcome the limitations of classical CARS, an improved variable selection strategy based on the RSA was proposed, termed DRSA. Compared to the original RSA, DRSA introduces three key strategies: dynamic exploration probability decay, exploration strength modulation, and randomized behavior switching.

(1) Exponential decay of exploration probability.

To smoothly transition from global exploration to local exploitation, DRSA applies an exponential decay function to control the probability of exploration behavior:


$$P_{explore}\left(t\right)=e^{\frac{-\text{λt}}{T_{max}}}$$

where *λ* is a decay rate parameter, *t* is the current iteration, and *T*_max_ is the total number of iterations. This design allows DRSA to perform global search in early iterations and switch to local fine-tuning in later stages.

(2) Exploration strength modulation.

A dynamic disturbance factor *ES* is introduced in the belly-walking behavior to enhance population diversity:


$$ES=2\times r\times \left(1-\frac{t}{T_{max}}\right),r\in \left\{-1,\;0,\;1\right\}$$

This factor is used to perturb individuals in the search space when simulating the belly-walking action:


$$x_{i,\;j}\left(t+1\right)=Best_{j}\left(t\right)\times x_{rj}\left(t\right)\times ES\times rand()$$

(3) Behavior-based position update mechanism.

Each iteration randomly selects one of four reptilian behaviors for each individual and dimension:

High walking:


$$x_{i,\;j}\left(t+1\right)=Best_{j}\left(t\right)-\eta_{i,j}\times \beta -R_{i,j}\times rand()$$

Belly walking:


$$x_{i,\;j}\left(t+1\right)=Best_{j}\left(t\right)\times x_{rj}\times ES\times rand()$$

Coordination hunting:


$$x_{i,\;j}\left(t+1\right)=Best_{j}\left(t\right)\times P_{i,\;j}\times rand()$$

Cooperation hunting:


$$x_{i,\;j}\left(t+1\right)=Best_{j}\left(t\right)-\eta_{i,\;j}\times \epsilon -R_{i,\;j}\times rand()$$

where


$$R_{i,\;\;j}=\frac{Best_{j}-x_{rj}}{Best_{j}+\epsilon }$$

$$P_{ij}=\alpha +\frac{x_{ij}-mean\left(x_{i}\right)}{Best_{j}\times \left(UB_{j}-LB_{j}\right)+\epsilon }$$

$$\eta_{i,j}=Best_{j}\times P_{i,j}$$

**Figure 1:**
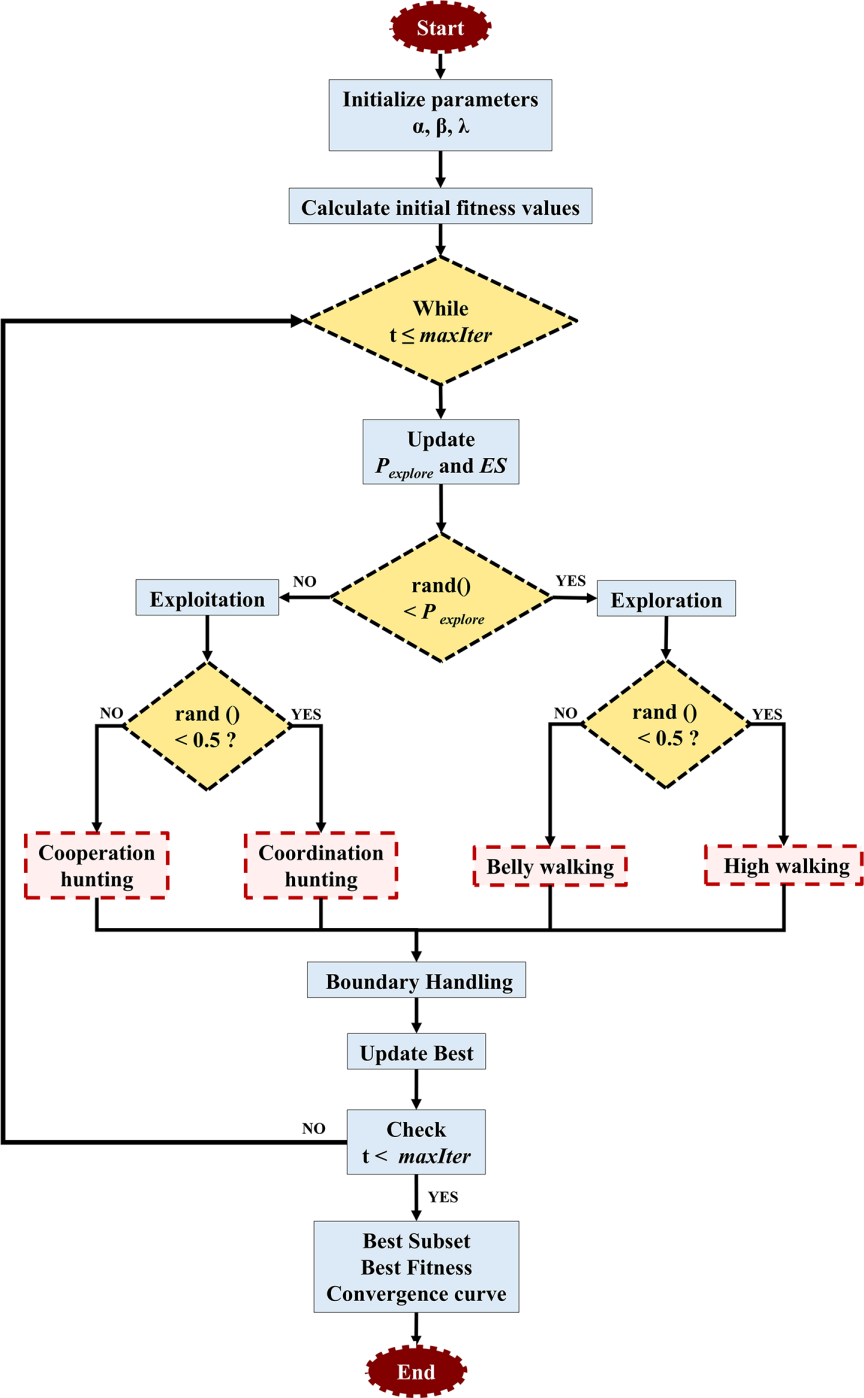
The dynamic behavior-based reptile search algorithm for feature selection.

This strategy enables DRSA to flexibly alternate between exploration and exploitation while preserving a balance between convergence accuracy and global search ability. The structures of this modified algorithm are illustrated in **Figure 1**. Compared with other RSA variants that rely on chaotic maps or simulated annealing (Abualigah et al., 2022; Elgamal et al., 2022; Khan et al., 2023), the DRSA-CARS adopts a behavior-driven strategy that is both adaptive and computationally efficient. This design helps the algorithm avoid local optima and remain stable during convergence. In the modified CARS framework, the DRSA-CARS module selects the optimal variable subset by minimizing the RMSECV of the PLS model. The selected bands from both methods were used to establish PLSR models with fixed parameters. The one exhibiting superior predictive performance was selected for subsequent analysis and modeling.

#### 2.5.3 Selection of spectral vegetation index

The relationship between chlorophyll content and VIs, widely regarded as a non-destructive and high-throughput method, has been extensively discussed in the context of precision agriculture for monitoring photosynthetic capacity and growth status (Bannari et al., 1995; Kandpal and Kumar, 2024). VIs, which combine reflectance information from the visible and infrared regions to extract biophysically meaningful indicators from HSI, are designed to maximize sensitivity to target traits while minimizing interference from non-target factors. In this study, the predictive power of VIs for plant growth status and phenotypes such as SPAD was not only supported by statistical performance but also rooted in physiological principles. The red edge (680–750 nm) and near-infrared (750–1,000 nm) spectral regions are particularly sensitive to chlorophyll concentration and internal leaf structure, respectively. VIs, such as the chlorophyll index red edge (CI rededge) and the normalized difference red edge index (NDRE), leverage these spectral regions to capture photosynthetic activity, thereby exhibiting strong correlations with SPAD values. Similarly, the green spectral region (540–560 nm), commonly used in the chlorophyll index with green (CI green), provides complementary information related to leaf pigments and enhances the sensitivity of VIs to chlorophyll dynamics. Therefore, chlorophyll-sensitive VIs, including CI green, CI rededge, NDRE, and modified simple ratio with red edge (MSRREG), were selected based on their physiological relevance and strong correlations with chlorophyll content (Qiao et al., 2022). In addition, classical VIs, including the normalized difference vegetation index (NDVI), enhanced vegetation index (EVI), optimized soil-adjusted vegetation index with red edge (OSAVIREG), and red difference vegetation index with red edge (RDVIREG), were incorporated to capture broader plant growth and physiological status (Qiao et al., 2022). Hence, eight VIs were selected to evaluate the relative chlorophyll content to assess the growth status of *L. rotata*, and the detailed VI calculation formulae are presented in **Table 2**. To determine the optimal band combination for VI calculation, all possible pairs among the red edge (680–750 nm), near-infrared (700–1,000 nm), and green (540–560 nm) bands were systematically evaluated. The pair that yielded the highest Pearson correlation coefficient with SPAD values was selected for each index.

**Table 2:** The vegetation indices utilized in this study.

Vegetation index	Formula	Illustration	References
Chlorophyll index with green (CI green)	$\text{CI green=}\frac{{\text{NIR}}_{871}}{{\text{G}}_{558}}-1$	Is used to assess the overall health of vegetation	Qiao et al. (2022)
Chlorophyll index red edge (CI rededge)	$\text{CI rededge=}\frac{{\text{NIR}}_{871}}{{\text{REG}}_{718}}-1$	Tracks the chlorophyll content changes for monitoring plant health and growth	Qiao et al. (2022)
Enhanced vegetation index (EVI)	$\text{EVI=}\frac{2{\text{.5×(NIR}}_{871}{\text{-R}}_{726})}{{\text{NIR}}_{873}{+6\times R}_{726}-7{.5\times B}_{450}+1}$	Sensitivity to low vegetation saturation and is used for analysis in high vegetation cover areas	Qiao et al. (2022)
Modified simple ratio with red edge (MSRREG)	$MSRREG=\frac{\frac{NIR_{871}}{REG_{718}-1}}{\sqrt{\frac{NIR_{871}}{REG_{718}+1}}}$	Estimates the photosynthetic efficiency of vegetation to monitor growth status	Wu et al. (2023b)
Normalized difference red edge (NDRE)	$NDRE=\frac{NIR_{871}-REG_{720}}{NIR_{871}+REG_{720}}$	Is used for assessing plant nitrogen content and chlorophyll	Qiao et al. (2022)
Normalized difference vegetation index (NDVI)	$NDVI=\frac{NIR_{871}-R_{720}}{NIR_{871}+R_{720}}$	One of the most common vegetation indices and is used for monitoring vegetation growth status	Qiao et al. (2022)
Optimized soil-adjusted vegetation index with red edge (OSAVIREG)	$\text{OSAVIREG=}\left(1 + 0.16\right)\left({\text{NIR}}_{873}{\text{-REG}}_{726}\right)\left({\text{NIR}}_{873}{\text{+REG}}_{726}+0.16\right)$	Reduces the influence of soil background through an improved algorithm	Qiao et al. (2022)
Red difference vegetation index with red edge (RDVIREG)	$\text{RDVIREG=}\frac{{\text{NIR}}_{873}{\text{-REG}}_{727}}{\sqrt{{\text{NIR}}_{873}{\text{+REG}}_{727}}}$	Focuses on changes of chlorophyll content and is often used to monitor growth and health	Qiao et al. (2022)

#### 2.5.4 Selection of textural features

In visible and infrared wavelength remotely sensed images, texture has provided information on independent spectral reflectance values and can improve the model accuracy (Hall-Beyer, 2017). In the HSI, texture features extracted from the spectrum image can capture the spatial variability of object surface information by combining spectral information with spatial structure, which has been widely employed to measure weight (Saif et al., 2023). The pixel’s gray value and its spatial relationship with neighboring pixels can be used to capture the local texture characteristics of the image (Wan et al., 2023). The gray-level co-occurrence matrix (GLCM) proved to be the most effective metric for assessing biomass and has been widely used to extract texture features (Kelsey and Neff, 2014; Zheng et al., 2019). Traditionally, texture features were extracted from each individual spectral band and resulted in high computational cost and substantial redundant information due to strong interband correlation. In this study, principal component analysis (PCA) was first applied to reduce dimensionality to extract representative texture features from hyperspectral images. Then, the first three principal components (PCs), accounting for the representative bands with the highest contribution to spectral variation, were reconstructed into spatial images. For each PC, the spectral band with the highest absolute loading weight was selected as the most representative band for texture extraction. This texture feature extraction method based on PCA not only reduces computational burden and minimizes redundant information but also retains essential spatial variation relevant to plant phenotypes. From each selected band image, four statistical texture features, including mean (MEA), contrast (CON), dissimilarity (DIS), and entropy (ENT), were calculated based on the GLCM method following the formulation proposed by Hall-Beyer (2017). The above textural features quantitatively describe the spatial arrangement of pixel intensities. Particularly, CON and DIS represent spatial variation and edge sharpness, ENT quantifies the randomness and complexity of the pixel arrangement, and MEA captures the average pixel intensity within the region of interest. Therefore, the aforementioned textural features are closely associated with plant architectural characteristics such as canopy complexity and leaf arrangement. Consequently, they serve as useful indicators of plant vigor and aboveground biomass. The extracted features were then used for subsequent model development.

#### 2.5.5 Model development and evaluation

In this study, classical supervised learning of PLSR and support vector regression (SVR) and neural networks of feedforward neural network (FNN) and convolutional neural network (CNN) were used to establish the prediction model to assess the SPAD and aboveground biomass of *L. rotata*, respectively. PLSR is the most prevalent method for establishing data matrices of *X* and *Y* through a linear multivariate model (Wold et al., 2001). SVR, a representative of supervised machine learning, aims to make sample points closely approximate the regression hyperplane as much as possible to handle regression problems (Zhang and O’Donnell, 2019). Both methods were extensively applied to the quality of herbal medicine (Li et al., 2023). Deep learning algorithms, renowned for their exceptional self-learning capabilities, are especially adept at processing high-dimensional spectral data, thereby enhancing prediction accuracy (Wang et al., 2023). Among deep learning models, the FNN, commonly used with the backpropagation learning algorithm, has been effectively utilized in various domains, including pattern classification, clustering, and regression (Shaik et al., 2020). Furthermore, CNN is particularly favored for evaluating traditional Chinese medicine by simultaneously performing multiple non-linear processing tasks to achieve a globally optimal prediction of the target variables (Wang et al., 2024). Therefore, the integration of DL and HSI should be further investigated to explore its potential as a non-destructive technique for estimating plant phenotypes and expanding the application of HSI in analysis.

The preprocessed data matrix was used to establish the PLSR, SVR, FNN, and CNN models based on their characteristic bands or selected features. In order to evaluate the generalization ability of the models, the data matrix was divided into training, validation, and test sets with a ratio of 7:2:1 by the function “cvpartition.” To comprehensively evaluate the performance of the prediction models, several statistical metrics were employed, including the coefficient of determination (*R*²), root mean square error (RMSE), and residual prediction deviation (RPD). *R*² was used to assess the proportion of variance in the observed data that can be explained by the model, with higher values indicating a better model fit. RMSE was calculated to quantify the average magnitude of prediction errors, with lower values indicating better predictive accuracy. Additionally, RPD, defined as the ratio of the standard deviation of the measured values to the RMSE, was adopted to assess the robustness and generalizability of the models. An RPD value between 1.5 and 2 was considered acceptable, and a value greater than 2 generally indicated a model with strong predictive ability. The above evaluation criteria were applied to the training, validation, and test datasets for each model, enabling a comprehensive comparison of the predictive performance among the PLSR, SVR, FNN, and CNN models. During data processing, MATLAB R2023b (MathWorks, Inc., USA) was used to establish the PLSR, SVR, FNN, and CNN models, and statistical data were calculated by GraphPad Prism version 8.3.0 (GraphPad Software Inc., San Diego, CA, USA).

## 3 Results

### 3.1 Statistical analysis of phenotypic characteristics

**Figure 2:**
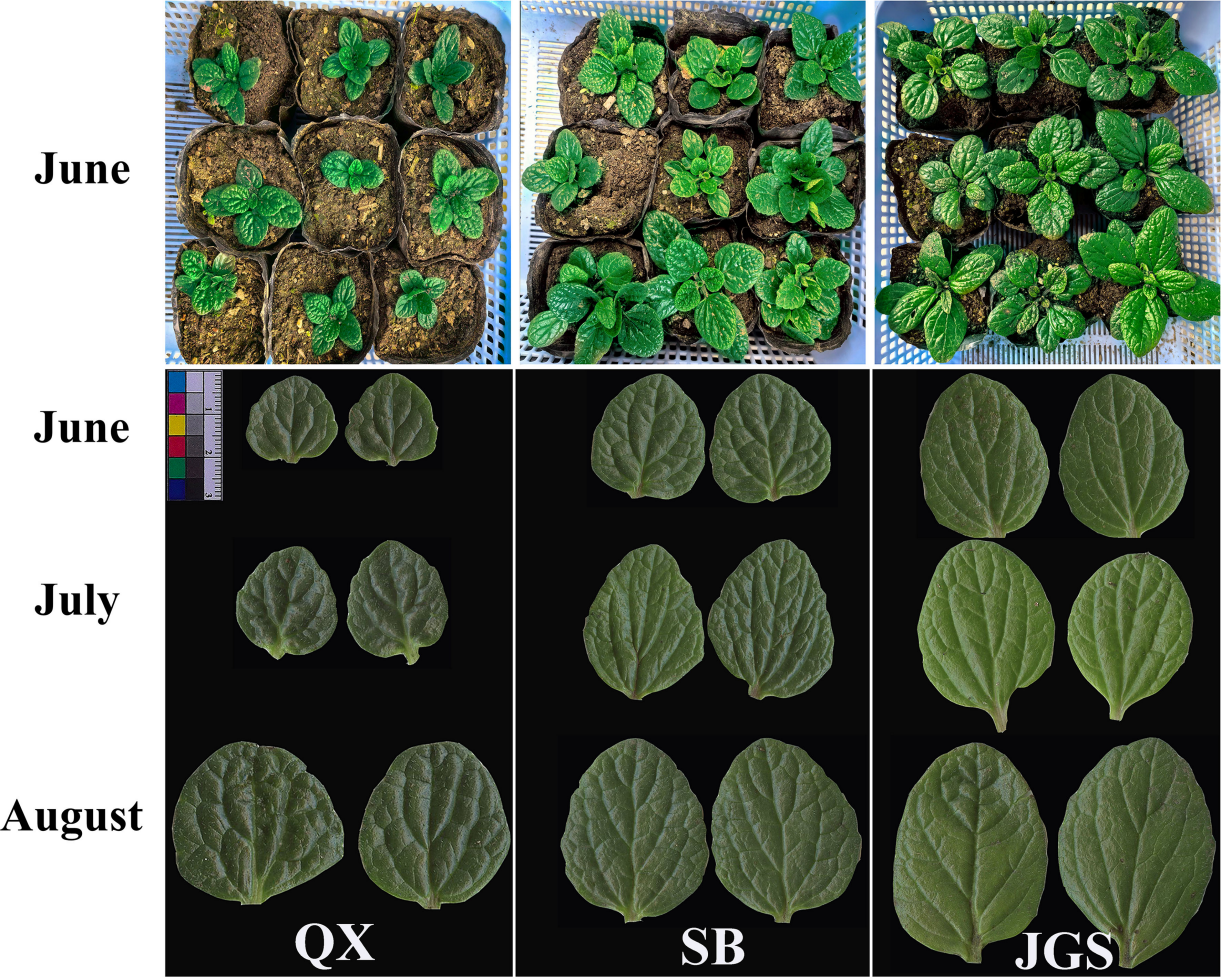
Phenotypic characteristics of *Lamiophlomis rotata* observed across three altitudes and three sampling periods.

The phenotypic characteristics of *L. rotata* are illustrated in **Figure 2**. Variations in leaf morphology among samples from QX, SB, and JGS are presented in the upper panel of the figure. Whole-plant images highlighted phenotypic differences associated with altitude. *Lamiophlomis rotata* grown at the high-altitude site QX exhibited a smaller overall size compared to those from SB and JGS. Notably, SB and JGS showed comparable growth performance. Scanned images of opposite leaves from all three sites (QX, SB, and JGS) at three growth stages are displayed in the lower panel of **Figure 2**. Over the growing season, all *L. rotata* samples displayed a progressive darkening of leaf color. Notably, the leaves from QX consistently exhibited the smallest area among all sites.

**Figure 3:**
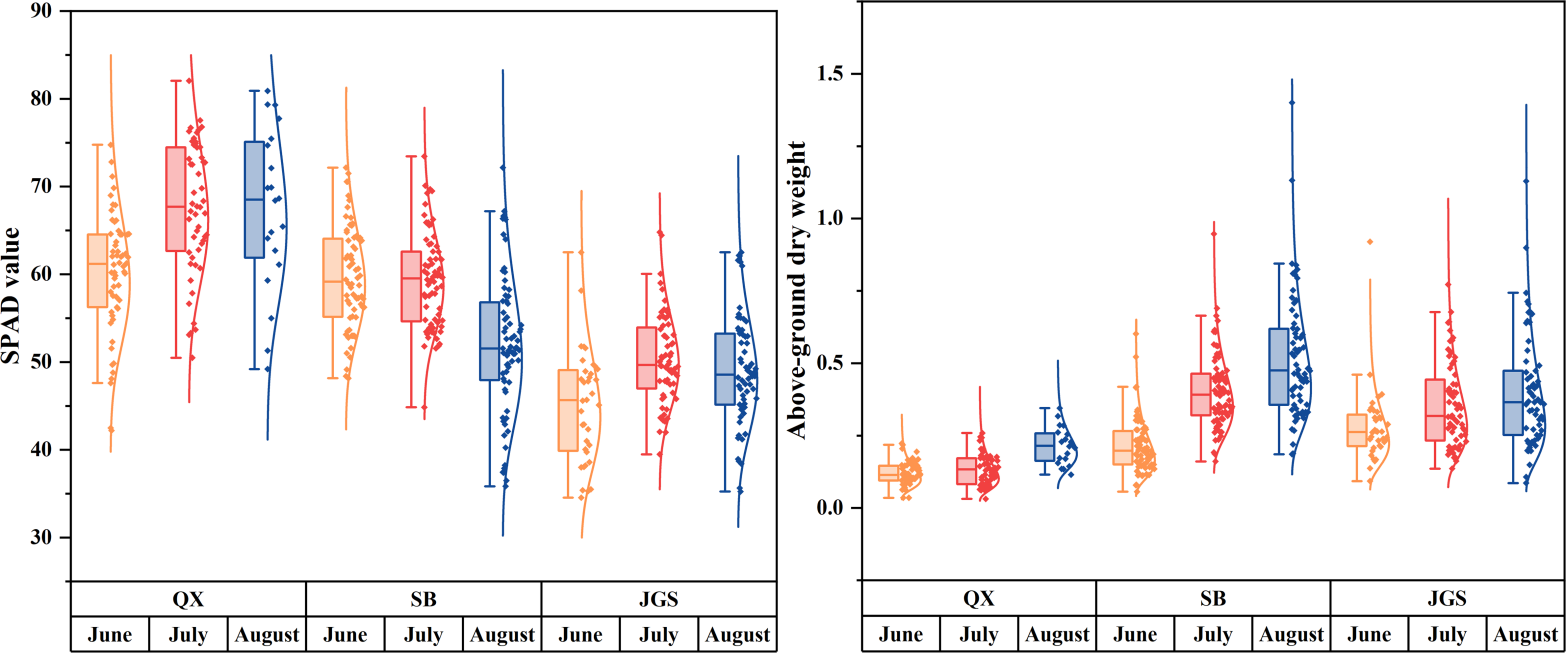
The range of reference values of SPAD and aboveground biomass.

Furthermore, statistical analysis was conducted to quantify differences in SPAD values and aboveground biomass. **Figure 3** summarizes the relevant parameters: SPAD values are shown on the left, while aboveground dry weights are presented on the right. SPAD values were the highest in samples from QX, followed by SB, and the lowest in JGS. Temporal fluctuations in SPAD values are attributed to differences in sampling times. Plants from QX maintained low and relatively stable aboveground biomass throughout the growing season. In contrast, plants from SB and JGS exhibited significantly higher and gradually increasing biomass over time. These morphological trends suggest that phenotypic plasticity may facilitate acclimation to lower-altitude environments. Therefore, the development of a rapid, non-destructive model for quality assessment is crucial to support the successful introduction and cultivation of *L. rotata*.

### 3.2 Raw and preprocessed spectral profile

**Figure 4:**
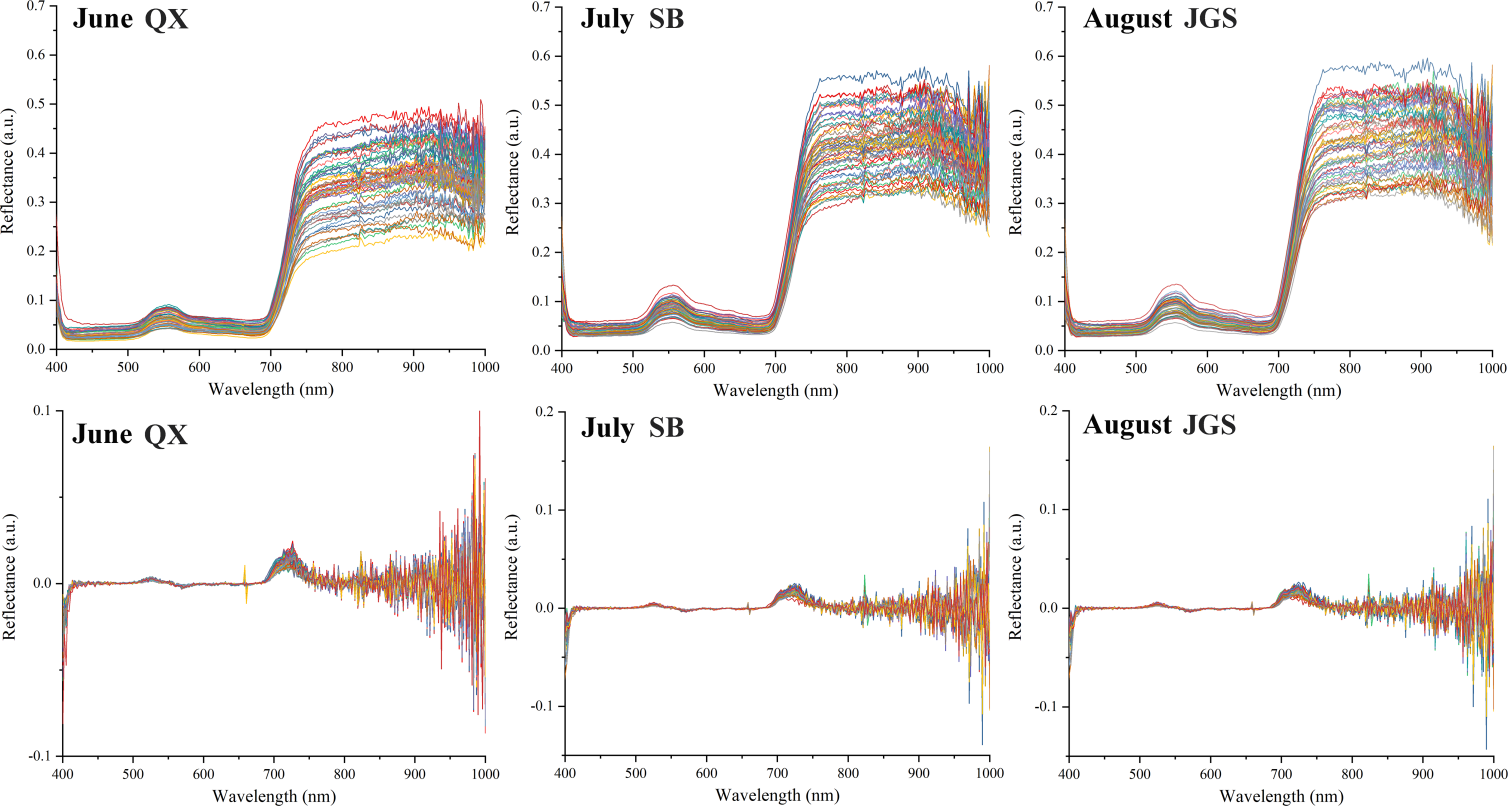
HSI spectra and the preprocessed by first-order derivative of *Lamiophlomis rotata* samples.

The spectral reflectance of all *L. rotata* samples was recorded across the 384~1,022-nm range, as shown in **Figure 4**. The raw spectral curves exhibited similar overall trends and shapes. However, reflectance differences were observed among the samples from the three altitudes at the same time point. The spectral curves from QX exhibited a narrower distribution range compared to those from SB and JGS. Specifically, reflectance values for *L. rotata* from QX were concentrated between 0.20 and 0.55. In contrast, the values from SB and JGS ranged from 0.25 to 0.60. The spectral curves from QX, SB, and JGS also displayed distinct absorption characteristics.

Therefore, in-depth spectral analysis is essential for the accurate assessment of plant growth status and biomass. To improve spectral interpretability and model performance, four preprocessing techniques, namely, SNV, MSC, SG, and FD, were applied to the raw spectra. As shown in **Table 3**, the FD method yields the best predictive performance, achieving the highest coefficient of determination (*R*²) and the lowest root mean square error (RMSE) for both SPAD and aboveground biomass. Based on these results, the FD preprocessed spectral data were selected for the subsequent modeling.

**Table 3:** The preprocessing method results for HSI based on the PLSR model.

Preprocessing methods	Quality attributes	*R* ^2^	RMSE	*R* ^2v^	RMSECV	*R* ^2p^	RMSEP
SNV	SPAD	0.5291	5.8130	0.6334	6.4327	0.6601	5.6906
	Aboveground biomass	0.3440	0.1503	0.2893	0.1493	0.2832	0.2298
MSC	SPAD	0.5291	6.4327	0.6334	5.8130	0.6601	5.6906
	Aboveground biomass	0.3440	0.1493	0.2893	0.1503	0.2832	0.2298
SG	SPAD	0.4898	6.6959	0.5409	6.3774	0.5524	6.4811
	Aboveground biomass	0.4341	0.1387	0.4389	0.1337	0.4562	0.2083
FD	SPAD	**0.6018**	**5.9154**	**0.6339**	**5.7490**	**0.6890**	**5.5899**
	Aboveground biomass	**0.4810**	**0.1328**	**0.3402**	**0.1465**	**0.4119**	**0.2101**

Bold values indicate the best performance values for each quality attribute (SPAD or Aboveground biomass) among the compared preprocessing methods.

### 3.3 Characteristic wavelength analysis

Based on the preprocessed hyperspectral data matrix, both the classical CARS and the improved DRSA-CARS methods were employed to identify informative spectral bands for predicting SPAD values and aboveground biomass. These feature selection approaches aim to reduce data redundancy while retaining key variables associated with the target traits. In the classical CARS method, feature weights are iteratively updated over 200 sampling runs. Important variables are selected based on their contributions to the PLS model, with the number of principal components constrained to a maximum of 10. The optimal subset is determined according to the lowest RMSECV. **Supplementary Figure S1** illustrates the variable selection process and results. For characteristic wavelength selection in SPAD, the lowest RMSECV was observed at the ninth iteration, resulting in 140 selected variables. Similarly, the optimal subset for aboveground biomass was identified at the 18th iteration and yields 106 variables. After eliminating duplicate indices, the final number of selected variables is shown in **Table 4**.

**Table 4:** The feature extraction results of the CARS and DRSA-CARS methods.

Feature extraction methods	RMSE	*R* ^2^	Number of selected features
CARS (SPAD)	6.9894	0.4632	140
CARS (aboveground biomass)	0.1626	0.3347	106
DRSA_CARS (SPAD)	**6.2113**	**0.5761**	**34**
DRSA_CARS (aboveground biomass)	**0.1478**	**0.4502**	**75**

Bold values indicate the best performance values for each quality attribute (SPAD or Aboveground biomass) among the compared feature extraction methods.

**Figure 5:**
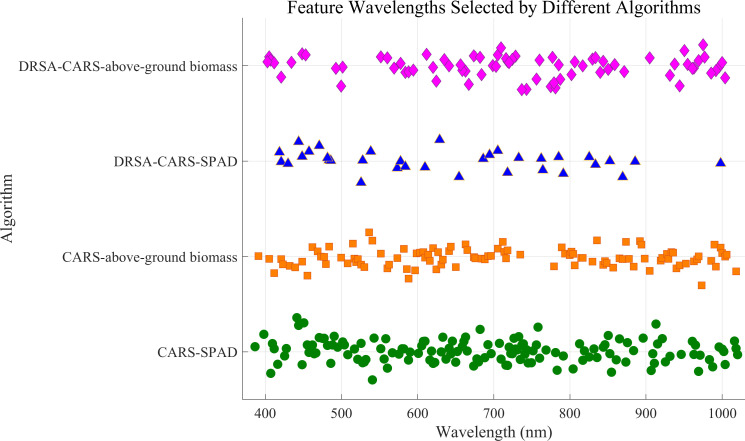
The feature wavelength selected by CARS and DRSA-CARS.

The CARS method was further integrated with DRSA to form the DRSA-CARS framework, which aims to enhance global search capability and stabilize feature selection. In this approach, the number of variables is adaptively adjusted, and dynamic behavioral strategies are employed during the search process (**Supplementary Figure S2**). As a result, 34 bands were selected for SPAD prediction and 75 for aboveground biomass (**Table 4**). **Figure 5** displays the spectral distribution of feature wavelengths selected by CARS and DRSA-CARS for both traits. Each point represents a selected wavelength. Their horizontal positions corresponded to specific wavelengths in the hyperspectral range of 380–1,020 nm. For both prediction tasks, fewer but more concentrated wavelengths were selected by the DRSA-CARS method compared to classical CARS. Specifically, a large number of bands selected by the CARS were broadly distributed across the visible and near-infrared (NIR) regions. In contrast, the DRSA-CARS variants yielded more compact subsets. These selected wavelengths were evenly distributed, with clearer clustering patterns, particularly in vegetation-sensitive regions such as 550–750 nm and 900–1,000 nm. These regions were associated with chlorophyll absorption and red-edge effects, suggesting that the DRSA-CARS method can better capture physiologically relevant spectral features. Overall, the visualization indicated that feature dimensionality was reduced and biological interpretability was enhanced by DRSA-CARS through its focus on informative spectral intervals. Hence, the feature wavelengths selected by the DRSA-CARS method were used for the following analysis.

### 3.4 Calculation of vegetation index

To enhance the physiological relevance and predictive power of VIs, multiple red-edge and near-infrared band combinations were explored. The final selected indices were constructed based on optimized wavelengths, particularly centered around NIR_871_–_873_ nm and REG_718_–_727_ nm, which lie within the key spectral domains associated with chlorophyll absorption, red-edge transition, and canopy structure sensitivity (**Table 2**). Notably, indices such as NDRE, NDVI, and RDVIREG utilized NIR_871_ or NIR_873_ in combination with REG_720_–_727_, which corresponds to the classical red-edge inflection zone. This region is known for its high sensitivity to chlorophyll concentration and photosynthetic activity, supporting its effectiveness in SPAD prediction. Moreover, NDRE and RDVIREG leveraged the contrast between NIR and red-edge reflectance to enhance the detection of subtle differences in leaf pigment levels, particularly under varying environmental stress conditions. In addition, indices such as EVI and OSAVIREG integrated both blue (B_450_) and red-edge (REG_726_) components, thereby not only capturing pigment content but also reducing soil background and atmospheric noise. Compared to traditional broadband indices, these indices showed correspondence to regions with well-documented links to plant biochemical and structural traits. These results supported the fact that integration of red-edge NIR-sensitive VIs with statistically optimized features may enable a robust and biologically meaningful prediction framework for chlorophyll-related traits in *L. rotata*. Therefore, the optimized VIs combined with the DRSA-CARS selected spectral bands were integrated into a new feature set for SPAD prediction in subsequent analysis.

### 3.5 Extraction of textural features

**Figure 6:**
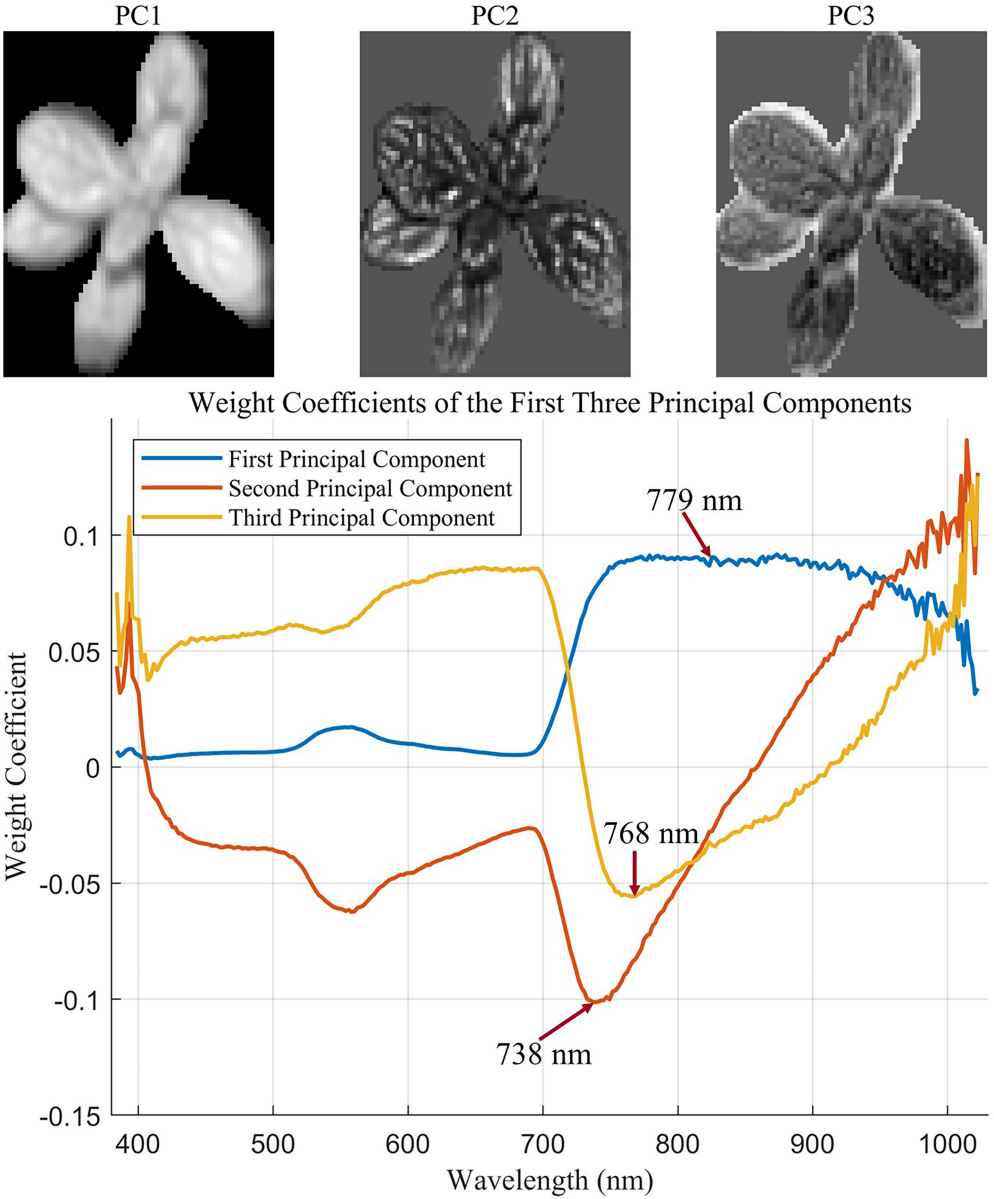
The textural feature extraction results (take sample no. 1 as an example).

The GLCM can be applied to extract texture information across the hyperspectral image. However, a large amount of redundant spectral information may be included when extracting texture features from all bands. To minimize the influence of irrelevant information, PCA was applied to the images from selected regions of interest for dimensionality reduction. The cumulative contribution of the first three principal components (PCs) was calculated to evaluate the proportion of total variance explained in the data. The wavelengths with the highest contributions to each principal component were identified based on the component loading coefficients. **Figure 6** displays the textural feature extraction results of sample no. 1. The cumulative contribution of the first three PCs reached 98.43%. The redundancy of texture features was significantly reduced after dimensionality reduction. In the images corresponding to the first three PCs, the leaf vein structure of *L. rotata* was clearly visible, enabling the identification of effective wavelengths (**Figure 6**). The bottom half of **Figure 6** shows the coefficient distribution for the first three PCs. The wavelength image corresponding to the maximum coefficient in each PC was selected as the effective wavelength image. In this analysis, the wavebands at 779 nm, 738 nm, and 768 nm were selected as effective wavelengths and were subsequently used for texture feature extraction. Texture features extracted from each principal component were compiled into a new feature vector. Specifically, four GLCM-based texture features were extracted from each PC, resulting in a total of 12 features (4 features × 3 PCs) per sample. The extracted texture feature vectors were then combined with the characteristic wavelengths identified by the DRSA-CARS algorithm. This newly fused data matrix was used to develop the prediction model for aboveground biomass.

### 3.6 Construction of the SPAD prediction models

**Figure 7:**
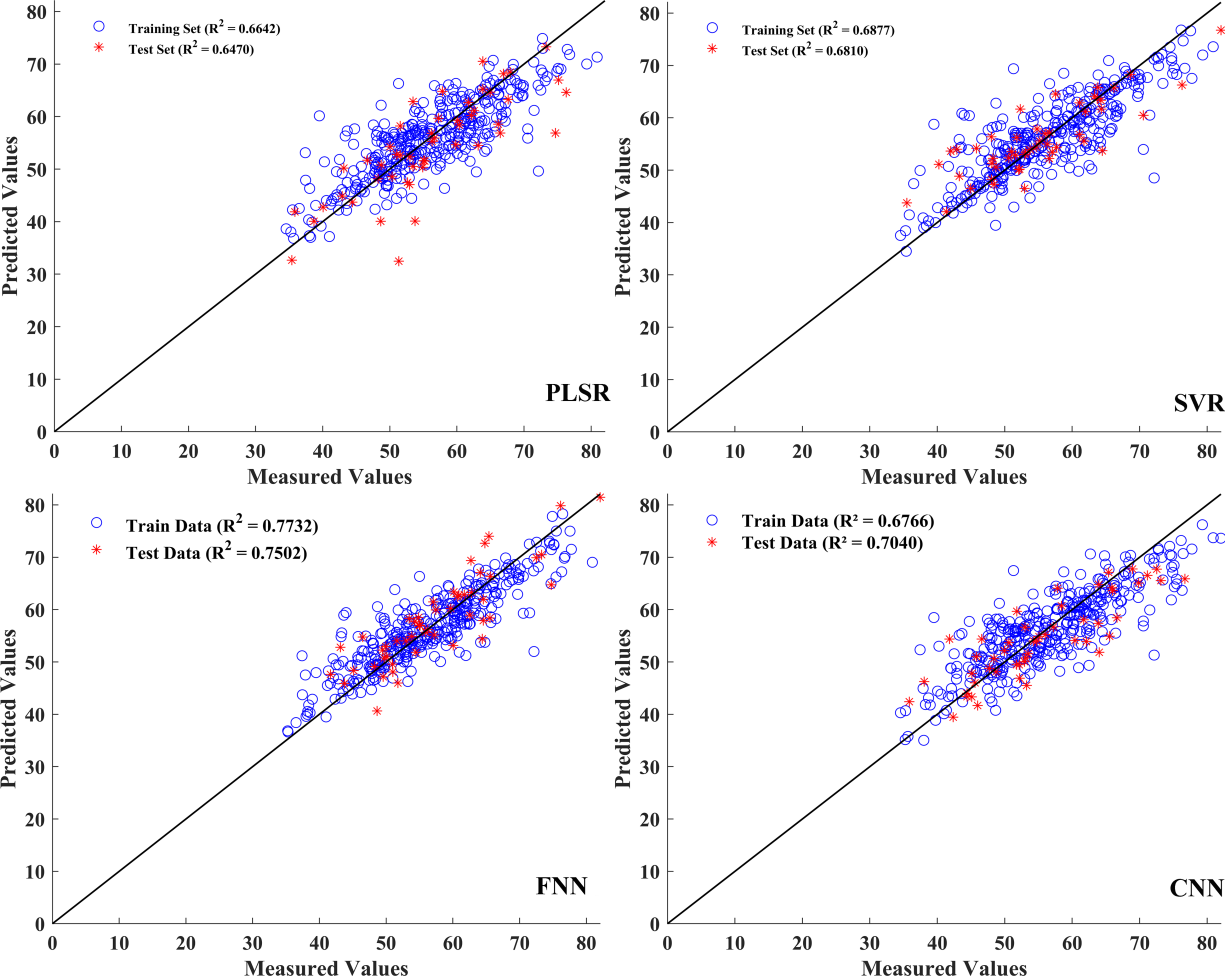
Prediction results of SPAD based on the PLSR, SVR, FNN, and CNN models.

In this process, the predictive performances of the four models, namely, PLSR, SVR, FNN, and CNN, were evaluated based on the single characteristic and the combination of vegetation index features. The single characteristic data matrix rows × columns were (508 × 34), and the fused data matrix rows × columns were (508 × 42). Both datasets were used to establish the SPAD prediction models before normalization. The performances of the prediction models based on the single and combined spectral and vegetation index information for SPAD estimation are presented in **Table 5**, with the corresponding model parameters summarized in **Supplementary Table S1**. In the prediction model established with features screened by the RSA-CARS method, the *R* ^2^ value of the training, verification, and test sets was approximately 0.65, among which the FNN model *R* ^2^ value shown in the test set was 0.6433 and had the best performance (**Table 5**). Compared with the single-feature established model, the model established with the dataset integrated with feature wavelength and vegetation index, the prediction performance of PLSR, SVR, FNN, and CNN models had improved. The *R* ^2p^ of the test set was improved by 1.3%, 7.0%, 10.7%, and 8.1%, respectively (**Table 5**). In the FNN model, the coefficients of determination were 0.7732 for the training set and 0.7502 for the test set, and the RPD was 1.9571, indicating good predictive performance for SPAD and reliable model predictions. In a previous study, an RPD of approximately 1.95 for SPAD similarly indicated that hyperspectral models can provide reliable, non-destructive estimates of leaf chlorophyll status (Riefolo and D’Andrea, 2024). Therefore, the RPD value of 1.9571 may not fully replace high-precision laboratory measurements. However, it shows that this model is useful for field applications, especially for comparative monitoring and tracking growth over time, where non-destructive assessment is important. **Figure 7** displays the prediction model results based on fusion features and vegetation index. The scatter plots between the measured and predicted values for the training set and the test set samples of the PLSR, SVR, FNN, and CNN models were built through the fusion of spectral and vegetation indices. The data in the training and test sets were relatively concentrated, especially for the FNN model, indicating that this model had a better detection effect. It can be seen that the combination of hyperspectral band information and vegetation index can improve the performance of the model.

**Table 5:** SPAD prediction results based on the characteristic bands and the fusion of vegetation indices.

Models	*R* ^2^	RMSE	*R* ^2v^	RMSECV	*R* ^2p^	RMSEP	RPD
PLSR characteristic band	0.6377	56.5985	0.6847	58.3853	0.6339	57.0362	0.1731
SVR characteristic band	0.6306	5.6402	0.7249	5.2756	0.6113	5.2263	1.6203
FNN characteristic band	0.6798	5.5000	0.6820	5.1654	0.6433	5.6243	1.5881
CNN characteristic band	0.6675	5.1877	0.5166	6.9849	0.6235	6.3991	1.6462
PLSR fusion vegetation index	0.6642	56.5970	0.6647	58.3787	0.6470	57.0201	0.1731
SVR fusion vegetation index	0.6877	5.0828	0.6758	5.9041	0.6810	5.1810	1.7885
FNN fusion vegetation index	**0.7732**	**4.4426**	**0.4622**	**7.4471**	**0.7502**	**4.5284**	**1.9571**
CNN fusion vegetation index	0.6766	5.2479	0.5914	6.1831	0.7040	5.2910	1.8565

Bold values indicate the best values within each column for comparison.

### 3.7 Construction of aboveground biomass prediction models

**Figure 8:**
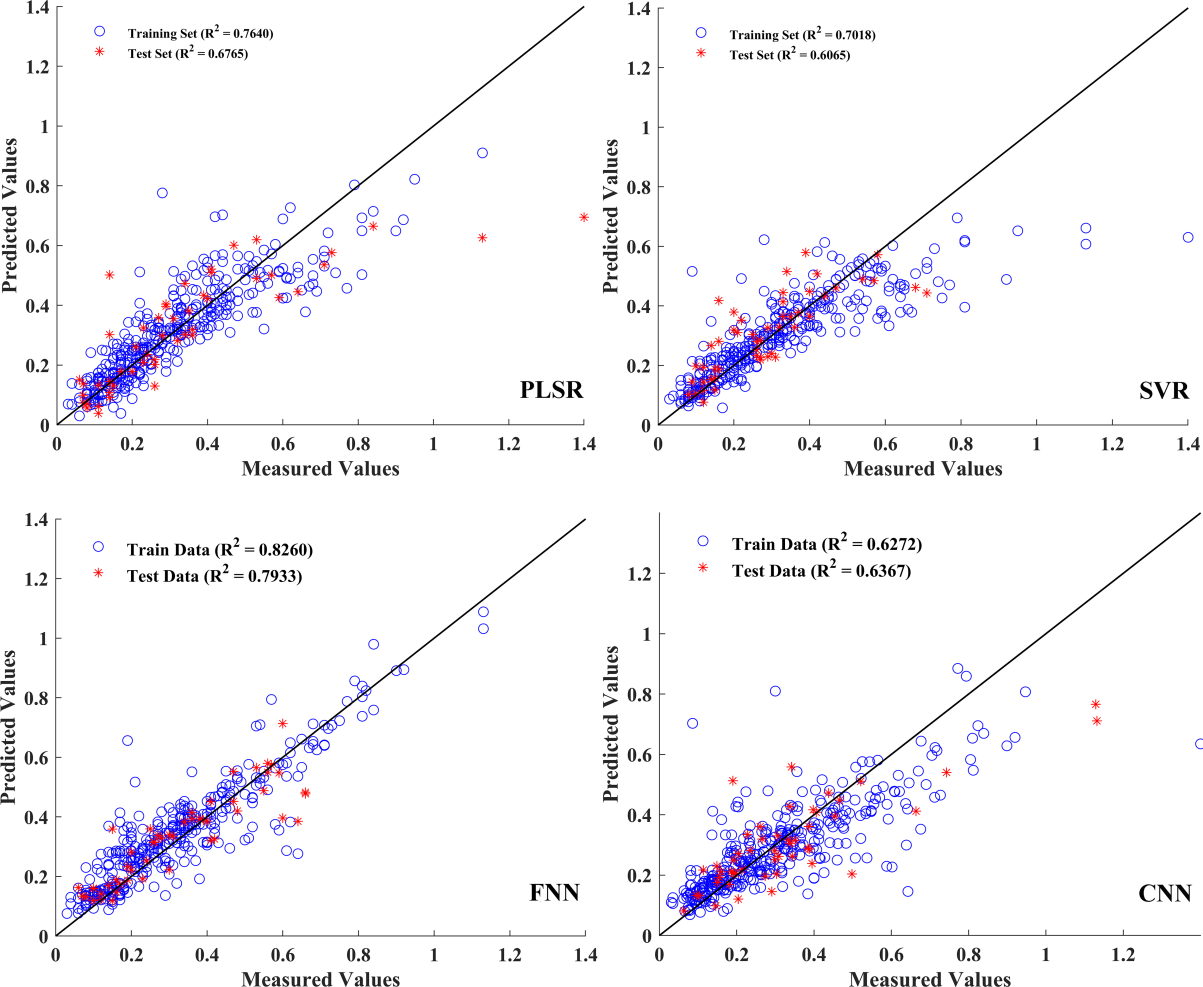
Prediction results of aboveground biomass based on the PLSR, SVR, FNN, and CNN models.

For the estimation of aboveground biomass, the model established by fusion features exhibited a better performance. **Table 6** shows the prediction model results, and the corresponding model parameters are provided in **Supplementary Table S2**. Specifically, in the single spectral model, built by 75 feature bands, the *R* ^2^ values of the training and test sets were approximately 0.5, and the RPD values were less than 1.5, implying the poor performance of these prediction models. The *R* ^2^ value of the training, validation, and test sets of the FNN model showed better performance than other models. Compared with the single spectra model results, the predictive performance of the model established by the fusion data was significantly improved. The *R* ^2p^ of the test set improved by 7.6%, 13.9%, 30.0%, and 22.7% in PLSR, SVR, ANN, and CNN, respectively (**Table 6**). Furthermore, the RMSEP and RPD values were 0.08 and 2.2, respectively, suggesting a reliable prediction performance of this model. **Figure 8** shows the relationships between aboveground biomass measured and estimated from the regression models established. In the fusion data model, the indices obtained from the FNN model were relatively close compared to those from the PLSR, SVR, and CNN models, suggesting that the FNN model had a reliable and accurate estimation.

**Table 6:** Aboveground biomass prediction results based on the characteristic bands and the fusion of textural features.

Models	*R* ^2^	RMSE	*R* ^2v^	RMSECV	*R* ^2p^	RMSEP	RPD
PLSR characteristic band	0.6171	0.7402	0.3607	0.8141	0.6004	0.7286	0.2119
SVR characteristic band	0.4930	0.1400	0.4473	0.1464	0.4671	0.1103	1.3837
FNN characteristic band	0.5074	0.1363	0.6052	0.1319	0.4937	0.1642	1.3815
CNN characteristic band	0.5465	0.1294	0.4432	0.1417	0.4098	0.1566	1.3149
PLSR fusion textural features	0.7640	0.0895	0.5866	0.1156	0.6765	0.1572	1.7139
SVR fusion textural features	0.7018	0.1074	0.6510	0.1163	0.6065	0.0948	1.6104
FNN fusion textural features	**0.8260**	**0.0831**	**0.8101**	**0.0904**	**0.7933**	**0.0819**	**2.1991**
CNN fusion textural features	0.6272	0.1175	0.6259	0.1121	0.6367	0.1288	1.6760

Bold values indicate the best values within each column for comparison.

## 4 Discussion

### 4.1 Phenotypic plasticity across altitude and growth state

The whole-plant images revealed phenotypic differences across the altitudes. QX plants were smaller than those from SB and JGS, suggesting that *L. rotata* cultivated at high altitudes faced significant environmental stress, such as cool temperature (**Table 1**), which may result in small and thick leaves. In contrast, *L. rotata* cultivated in SB and JGS showed better growth. *Lamiophlomis rotata* picked from SB and JGS consistently had the largest leaves. This suggests that both altitudes provided more favorable growing conditions than QX, leading to improved growth and development. Additionally, lower altitudes were associated with a significant increase in leaf length, indicating altitude-related plasticity in leaf development. Previous research has demonstrated that leaf morphology is regulated by environmental factors such as temperature and irradiance and that high-altitude conditions tend to restrict leaf expansion due to cold and UV stress (Wu et al., 2024).

The observed fluctuations in SPAD values across different sampling times suggested seasonal changes in chlorophyll concentration and photosynthetic capacity in *L. rotata*. *Lamiophlomis rotata* from QX always exhibited the darkest green leaves. This observation may be explained by stronger UV radiation at higher altitudes, which is known to promote the synthesis of photosynthetic pigments as a protective response to oxidative stress (Ren et al., 2010). Notably, the phenotypic differences observed in leaf coloration and morphology were consistent with the statistical patterns. SB plants showed moderate leaf color and stable SPAD values, while JGS plants had the lightest leaf color, likely indicating lower SPAD values and photosynthetic efficiency. These SPAD variations suggested varying levels of physiological adaptation to local environments, and elevation played a significant role in shaping the phenotypic diversity of *L. rotata.*

Similarly, the biomass of QX remained low and relatively stable from June to August, indicating limited vegetative growth throughout the season. In contrast, *L. rotata* from SB and JGS displayed significantly higher aboveground biomass. In both sites, biomass showed a general increasing trend from June to August. *Lamiophlomis rotata* cultivated in SB reached the highest biomass values by August, suggesting that these environmental conditions were more favorable for biomass accumulation. The statistical patterns of biomass accumulation aligned well with the phenotypic differences previously observed in plant stature, and these results indicated that elevation plays a significant role in shaping the phenotypic diversity of *L. rotata.* The morphological and physiological observations above suggested that phenotypic plasticity has occurred during acclimation to a lower altitude. However, we did not quantify the relationship between pharmacopeial marker compounds and medicinal quality, nor did we characterize the metabolic and molecular changes associated with phenotypic plasticity in *L. rotata*. Therefore, future work should integrate phytochemical profiling with molecular analyses. This approach will quantify links between pharmacopeia markers and bioactivity and elucidate the physiological and molecular mechanisms underlying altitude acclimation in *L. rotata*.

### 4.2 Spectral feature selection and characterization

The spectral signature of plants contained extensive details that reflected their growth status (Rehman et al., 2020). The observed differences in spectral reflectance across altitudes suggested that HSI was an effective tool for monitoring the growth condition of *L. rotata* in real time. The superior performance of the FD preprocessed spectra was likely attributed to its ability to capture reflectance slopes and amplify subtle spectral variations (Hou et al., 2024). In particular, the spectral region from 680 to 700 nm, which showed enhanced separability under FD processing, corresponded to the red-edge absorption domain associated with chlorophyll content. This region included a characteristic absorption peak near 680 nm (Xiao et al., 2022). This peak aligned with the measured differences in SPAD values among samples collected from different altitudes. Therefore, an appropriate preprocessing method used in HSI data was considered valuable for improving not only prediction accuracy but also their biological interpretability. This finding supported the application of HSI in plant growth assessment and biomass estimation.

Genetic algorithms and the Least Absolute Shrinkage and Selection Operator (LASSO) are two commonly used feature selection methods. The former explores the combinatorial search space stochastically but often requires substantial computation, while the latter provides efficient linear shrinkage but may yield biased coefficient estimates. In contrast, DRSA-CARS combines rule-based attribute reduction with adaptive sampling, enabling efficient spectral dimensionality reduction and the selection of informative wavelengths closely associated with plant physiological traits. These characteristic bands were primarily located in regions known for red-edge transition and near-infrared reflection, which are highly responsive to chlorophyll content, leaf structure, and biomass accumulation (Zhang et al., 2022b). Compared with traditional wavelength selection methods, the use of DRSA ensured global optimization and minimized redundancy, thereby increasing the speed and efficiency of feature selection (Khan et al., 2023). While DRSA improves search efficiency, its performance in extremely high-dimensional or noisy environments may be suboptimal, suggesting the need for adaptive mechanisms to further enhance its exploration and exploitation balance.

The VIs in this study were constructed by making full use of red-edge and near-infrared band combinations to enhance physiological relevance. Optimized indices such as NDRE, RDVIREG, and OSAVIREG could capture chlorophyll-related variation by leveraging reflectance differences in pigment-sensitive regions (Zhang et al., 2022c). Some selected bands (e.g., REG726 and REG727) were not included in the DRSA-CARS result. However, the strong physiological significance was supported by existing knowledge of chlorophyll absorption and red-edge positioning. The combination of biologically relevant VIs and statistically selected wavelengths provided a solid foundation for modeling.

In addition to spectral features, textural information extracted from hyperspectral images contributed meaningfully to biomass prediction. In this study, PCA was applied to reduce redundancy in the GLCM texture features extracted from each spectral band. The principal components with the highest variance explained were used to identify representative wavelength images, from which texture features were calculated. This approach retained structural patterns such as leaf veins while avoiding information overload, allowing the model to capture canopy-level spatial heterogeneity (Fan et al., 2024). However, the use of PCA for dimensionality reduction prior to texture extraction may lead to the loss of spatial detail or distortion of original image features. To address this limitation, future studies could explore 3D texture analysis methods to jointly preserve spectral and spatial integrity to achieve a better balance between interpretability and computational efficiency.

### 4.3 Integrative strategy for predicting the target variable

In this study, spectral, VIs, and texture features were combined within a unified modeling framework to predict SPAD and aboveground biomass. As anticipated, deep learning models outperformed traditional machine learning algorithms, reflecting their capacity to capture complex non-linear relationships from diverse input features. Among the deep learning models, the FNN consistently delivered the highest predictive accuracy for both traits, outperforming the CNN. This outcome was closely associated with the nature of the input data. Instead of employing spectral imagery, the models were provided with feature vectors that had undergone extensive preprocessing. These vectors were derived from mean spectra, first-derivative spectra, selected wavelengths, VIs, and GLCM texture metrics, which together substantially altered the original spatial dependencies. Although the preprocessing effectively isolated informative variables and reduced dimensionality, it inevitably removed the majority of pixel-level spatial dependencies (Zhao et al., 2025). However, CNN architectures relied on local spatial correlations to learn hierarchical representations. The loss of this spatial context in our data substantially diminished their performance advantage (Paoletti et al., 2019). In contrast, the FNN was well suited to compact, high-dimensional tabular inputs, effectively capturing non-linear patterns without relying on spatial neighborhood information. This contrast likely reflected both the FNN’s compatibility with the structured multi-source features and the reduced spatial context that constrained CNN performance, underscoring the importance of tailoring model architecture to the feature characteristics in hyperspectral trait prediction.

Interestingly, the prediction models developed with feature fusion improved the accuracy for SPAD and aboveground biomass by 11% and 30%, respectively. For SPAD, the prediction model integrated selected spectral bands with VIs that predominantly characterized leaf spectral properties, which may account for the relatively limited performance gain from feature fusion. In contrast, the biomass prediction model combined selected spectral bands with texture features extracted to capture spatial structural information. Texture features characterized canopy structural attributes, such as leaf arrangement, coverage, and gap distribution, that were directly associated with aboveground biomass (Dhakal et al., 2023). This relationship likely explains the greater performance improvement observed after fusion. While the fused feature model demonstrated promising performance, the relative importance of different feature types was treated as temporally static. However, plant phenotypic and physiological traits undergo dynamic changes across developmental stages, which can alter the predictive relevance of different hyperspectral features (Aasen et al., 2018). For instance, spectral reflectance tends to be more sensitive to pigment changes during early vegetative growth, while VIs often correlate more strongly with biomass accumulation and senescence traits in later stages. Overall, although the feasibility of fused feature modeling was confirmed in this study, the lack of temporal adaptation in feature contribution may limit its scalability. In future work, stage-aware modeling schemes should be incorporated to exploit the evolving predictive value of different hyperspectral feature domains.

In this study, hyperspectral data of *L. rotata* were acquired under controlled indoor conditions and combined with machine learning methods to establish a baseline framework for estimating SPAD and aboveground biomass. However, illumination variability and complex terrain, which are characteristic of alpine environments, represent major challenges for the deployment of this technology. The former introduces shadowing and bidirectional reflectance distribution function (BRDF) effects. Aasen et al. (2015) recommended controlling these effects through radiometric calibration using reflectance panels and a downwelling irradiance sensor, combined with near-noon flights. The latter causes fluctuations in ground-sampling distance and geometric quality along flight lines. These effects can be mitigated by terrain-following flight plans, high forward/side overlap, and DEM-assisted orthorectification and 3D reconstruction (Maes, 2025). Building on these considerations, a UAV-DL pipeline was established for wild *L. rotata* in our previous research (Ding et al., 2023). In that study, an identification precision of 89% was achieved on UAV orthomosaics using Mask R-CNN, and plot-scale counts and yield estimates were obtained. Accordingly, the integration of hyperspectral sensing with UAVs and deep learning for monitoring growth and bioactive compounds in wild *L. rotata* is currently underway. These efforts will support the standardized, routine deployment of hyperspectral technology for habitat monitoring of wild medicinal plants in high-altitude areas.

## 5 Conclusion

*Lamiophlomis rotata* samples were cultivated at three altitudes, and the time-series phenotype data were collected by HSI. Spectra and phenotypic data were selected as the research objects to monitor growth status and aboveground biomass of cultivated *L. rotata*. After spectrum preprocessing, feature band screening, and vegetation index and texture feature extraction, the selected feature band was fused with vegetation index and texture feature, and a prediction model of single feature input and multi-feature fusion input was constructed. The prediction effect and generalization ability of the model were compared. 1) The original spectrum was pretreated using the SNV, MSC, SG, and FD methods, and FD was the optimal method based on the fixed-parameter prediction model performance of PLSR. 2) Compared with CARS, the band features extracted by DRSA-CARS could reduce collinearity to obtain fewer and more efficient features. 3) The FNN prediction model constructed by integrating feature wavelength with vegetation index showed the best prediction performance for SPAD monitoring, and the coefficient of determination and the value of RPD in the test set were 0.7502 and 1.9571, respectively. 4) In the predictive models of biomass, FNN also exhibited optimal performance based on the fusion data of feature wavelength and texture features, and the coefficient of determination and the value of RPD in the test set were 0.7933 and 2.1991, respectively. The above studies show that models using the characteristic wavelength of HSI that fused features of vegetation index and texture characteristics performed better than those based on single features. This study verifies that the fusion features enhanced the prediction accuracy and improved the model performance by the spectral and spatial information collected from HSI. This also reveals the potential of combining multi-temporal HSI data with deep learning to enable dynamic growth monitoring and scalable applications in precision herb cultivation.

## Data Availability

The raw data supporting the conclusions of this article will be made available by the authors, without undue reservation.

## Supplementary material

Supplementary Figure 1 The result of feature bands screening by CARS.

Supplementary Figure 2 The process of feature band screening by DRSA-CARS algorithm.

Supplementary Table 1 Hyperparameters and settings of PLSR, SVR, FNN, and CNN models for SPAD prediction.

Supplementary Table 2 Hyperparameters and settings of PLSR, SVR, FNN, and CNN models for above-ground biomass prediction.
