# Supplementary figures and images for: Non-destructive estimation of SPAD and biomass in Lamiophlomis rotata using hyperspectral imaging and deep learning with DRSA-CARS feature selection
Source: Front Plant Sci. 2025 Sep 18;16:1640779. doi: 10.3389/fpls.2025.1640779 (PMC12488700; doi:10.3389/fpls.2025.1640779)

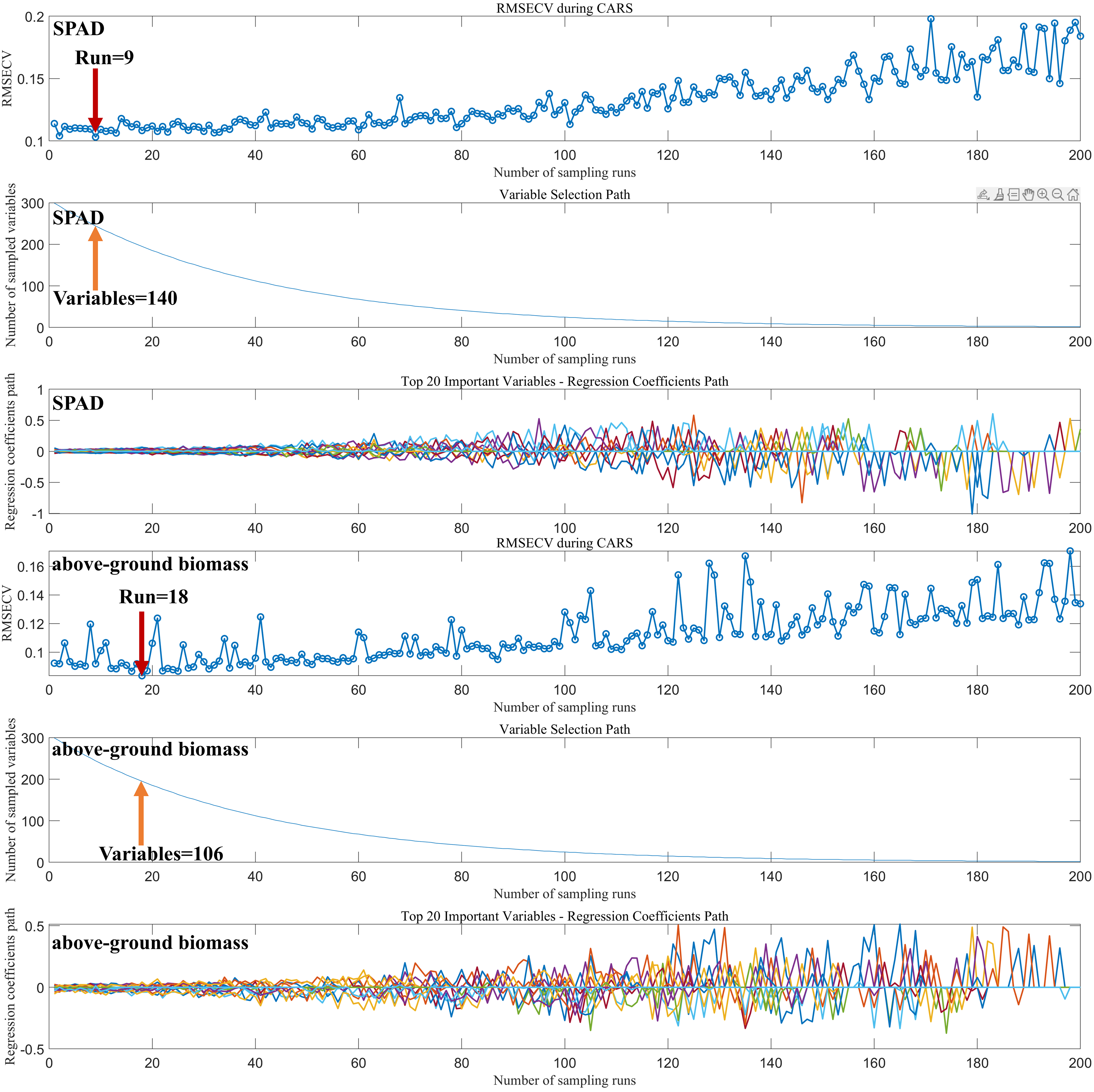

Supplement: Supplementary Figure 1 — The result of feature bands screening by CARS. [file Image1.tif]

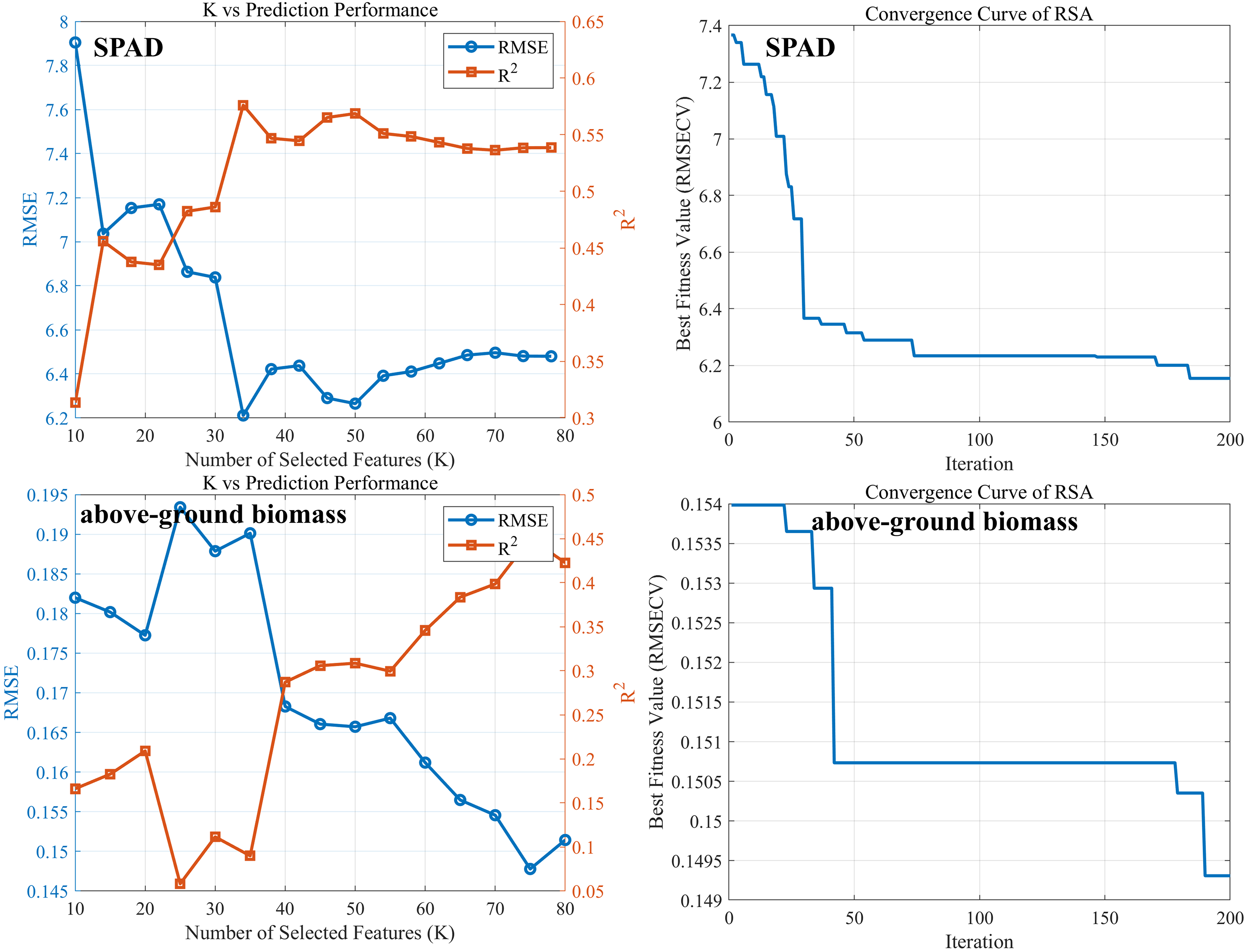

Supplement: Supplementary Figure 2 — The process of feature band screening by DRSA-CARS algorithm. [file Image2.tif]
